# Commentary: Association Between Alendronate Use and Hip Fracture Risk in Older Patients Using Oral Prednisolone

**DOI:** 10.3389/fnagi.2017.00357

**Published:** 2017-11-07

**Authors:** Xiang-Dong Wu, Ke-Jia Hu, Wei Huang

**Affiliations:** ^1^Department of Orthopaedic Surgery, The First Affiliated Hospital of Chongqing Medical University, Chongqing, China; ^2^Department of Neurosurgery, Massachusetts General Hospital, Harvard Medical School, Boston, MA, United States; ^3^Department of Microsurgery, Huashan Hospital, Fudan University, Shanghai, China

**Keywords:** bisphosphonates, prednisolone, hip fracture, atrial fibrillation, cardiovascular comorbidities

Axelsson et al. (2017) investigated the efficacy of alendronate for prevention of hip fracture among older patients treated with oral prednisolone, and concluded that “alendronate treatment was associated with a significantly lower risk of hip fracture over a median of 1.32 years.” Strengths of this study include a large sample size of patients with relative homogeneity and using a multivariable Cox model to control for potential confounders. Furthermore, the authors acknowledged the limitations of their work. Their efforts are significant and should be applauded. However, the readers should place the findings within wider clinical milieu, bearing in mind the other important outcomes during bisphosphonate treatment particularly cardiovascular events.

The association between increased risk of atrial fibrillation (AF) and bisphosphonates use has been reported a decade ago (Black et al., 2007). In 2008, the US Food and Drug Administration (FDA) announced that there was no significant risk of AF associated with the use of these drugs, but it would continue to monitor. Since then, many studies have been conducted to explore the relation between bisphosphonates and adverse cardiovascular outcomes, and conveyed contradictory findings. A meta-analysis reported (Sharma et al., 2013) a significantly higher risk of the AF which requiring hospitalization, other studies had reported increased incidences of acute ischemic stroke, congestive heart failure, and acute myocardial infarction (Wang et al., 2016).

AF is associated with higher risk of cardiovascular mortality, stroke, ischaemic heart disease and heart failure, and the association is significantly stronger in females than in males (Emdin et al., 2016). The present study enrolled elder patients with a mean age of nearly 80, more than half were female and many of them had cardiovascular comorbidities. Therefore, deaths due to cardiovascular disease should be concerned.

In summary, the study did not evaluate all the safety issues of bisphosphonates treatment in details, including the patients' mortality rate and the possible causes in the cohort. Considering bisphosphonates are very commonly used medications, and more and more indications are coming up, and majority of these patients are older and tend to have concomitant cardiac conditions, clinicians should be careful in selecting patients when using glucocorticoid in combination with bisphosphonates, especially for patients at high risk of AF—those who are older, have concomitant cardiac conditions or have a history of cardiac events—should be more closely monitored. This potential risk of cardiovascular events should be weighed against the reduction in the risk of hip fracture.

## Author contributions

X-DW, Contributed substantially to conception and design; drafted the article; gave final approval of the version to be published; agreed to act as guarantor of the work. K-JH, Contributed substantially to draft the article; gave final approval of the version to be published; agreed to act as guarantor of the work. WH, Contributed substantially to conception and design; revised it critically for important intellectual content; gave final approval of the version to be published; agreed to act as guarantor of the work.

### Conflict of interest statement

The authors declare that the research was conducted in the absence of any commercial or financial relationships that could be construed as a potential conflict of interest.
